# Comparative Effects of Hormone Replacement Therapy and Exercise on Bone Health in Postmenopausal Women: A Systematic Review

**DOI:** 10.7759/cureus.99210

**Published:** 2025-12-14

**Authors:** Marcela Treviño, Payton Leiker, Sainamitha R Palnati, Saajan Bhakta

**Affiliations:** 1 Medical School, Kansas College of Osteopathic Medicine, Wichita, USA; 2 Medical Affairs, Kansas College of Osteopathic Medicine, Wichita, USA

**Keywords:** bone mineral density (bmd), fracture risk, gynaecology and obstetrics, hormone replacement therapy (hrt), osteoporosis, postmenopausal, preventive practices, weight-bearing exercise therapy

## Abstract

Postmenopausal women are at increased risk of bone loss and fractures. This review compares hormone replacement therapy (HRT) and weight-bearing exercise in their ability to preserve bone mineral density (BMD), a key factor in osteoporosis prevention.

A systematic review of HRT and weight-bearing exercise therapy in postmenopausal women was conducted from February 24, 2025, to April 5, 2025, across six electronic databases. A full-text screening was completed by two independent reviewers following the Preferred Reporting Items for Systematic Reviews and Meta-Analyses (PRISMA) guidelines. Six studies were included according to the inclusion criteria, such as publication date, study format, intervention type, primary outcome, and follow-up duration. Data was extracted using Microsoft Excel (Microsoft Corp., Redmond, WA, USA), and a risk-of-bias assessment was completed.

Postmenopausal women may benefit from HRT, as there were greater improvements in BMD than exercise therapy alone and a decreased risk of fracture at sites such as the hip and vertebrae. Exercise interventions, particularly resistance-based or mixed-loading programs, also demonstrated improvements in BMD, although results varied depending on the type and intensity of exercise. Findings were varied and not uniformly superior to either intervention alone. There is also limited evidence evaluating the possible additive benefits of combined therapy. Additionally, discontinuing HRT after beginning it resulted in a decline in BMD, suggesting discontinuation is non-neutral.

Both HRT and weight-bearing exercise therapy were associated with improvements in BMD in postmenopausal women, with HRT resulting in greater increases than exercise alone. Exercise remains an important non-pharmacologic strategy, and combination therapy may provide an additive benefit, particularly at high-risk fracture sites such as the lumbar spine and hip, although further research is needed to clarify these effects.

## Introduction and background

There are roughly 82 million women in the United States who are in perimenopause, menopause, or postmenopause [1]. Studies have shown that this cohort of women lacked education on and desired more information about what their body was going through and how to manage it [2]. Menopause is defined as the cessation of menstruation for 12 consecutive months due to the decline in ovarian function and estrogen production. It typically occurs at a median age of 51 years in the United States, with approximately 1.3 million women transitioning into menopause annually [3]. Vasomotor symptoms such as hot flashes, urogenital changes, and a heightened risk of long-term health complications, including osteoporosis and cardiovascular disease, often accompany this shift [4,5].

Postmenopausal estrogen deficiency accelerates bone turnover, increasing bone resorption and resulting in a measurable loss of bone mineral density (BMD) [3,6]. Bone loss can begin before menopause and continues at a rate of 3% to 5% annually in the first five to seven years postmenopause, significantly increasing the risk of fragility fractures [3,6]. Osteoporosis represents a significant health concern among postmenopausal women, who are at the highest risk for accelerated bone loss and fragility fractures [3,7].

Hormone replacement therapy (HRT) provides the greatest bone-protective benefit when initiated before age 60 or within 10 years of menopause onset, consistent with the “timing hypothesis” [8,9]. Earlier initiation, especially within the first few years after menopause, may provide the greatest symptomatic and bone-related benefit. HRT is also currently the most effective intervention for managing the vasomotor symptoms and genitourinary syndrome of menopause and has also demonstrated efficacy in preserving BMD and reducing fracture risk [3,8]. However, HRT use has declined due to concerns regarding adverse effects, including breast cancer, stroke, and venous thromboembolism (VTE), though these risks vary significantly by formulation and route of administration [8,10]. For example, transdermal estradiol is associated with a lower risk of VTE and stroke compared to oral estrogen, and micronized progesterone carries a lower breast cancer risk than synthetic progestins [4,8].

For women who have contraindications or choose not to use HRT, there are alternative strategies such as selective serotonin reuptake inhibitors (SSRIs), neurokinin-3 receptor antagonists, and gabapentin to offer relief from vasomotor symptoms, but there is a lack of evidence for skeletal protection [11,12]. Lifestyle interventions, particularly weight-bearing and resistance exercise, have demonstrated efficacy in preserving BMD and mitigating fall risk in postmenopausal women [7,13]. Skeletal mass is largely influenced by mechanical loading, and impact loading exercise has been shown across several meta-analyses to have statistically significant effects on BMD in postmenopausal women. Additionally, regular exercise increases muscle strength and improves balance, which reduces fall risk and, therefore, reduces fracture risk. Another study determined that weight-bearing exercise less than three times per week was associated with increased risk of falls and hip fractures. Existing reviews have typically evaluated HRT or exercise in isolation, with few contemporary analyses directly comparing their effects, highlighting the need for an updated synthesis. This systematic review aims to synthesize existing literature comparing the effects of HRT and weight-bearing exercise on BMD and fracture risk in postmenopausal women. Both interventions have documented benefits on bone health, but comparative evidence evaluating their relative effectiveness remains limited. This review seeks to inform individualized treatment strategies that optimize skeletal health while balancing patient preference and risk profiles.

## Review

Methods

This was a systematic review following the Preferred Reporting Items for Systematic Reviews and Meta-Analyses (PRISMA) guidelines and criteria. The review scope and eligibility criteria were defined a priori using the population, intervention, comparator, outcome, and study design (PICOS) framework (Table 1).

**Table 1:** PICOS Criteria Naturally postmenopausal women are defined as women who have undergone the permanent cessation of menses for ≥12 consecutive months without other pathologic or surgical causes. PICOS: population, intervention, comparator, outcome, and study design; HRT: hormone replacement therapy; BMD: bone mineral density

Parameter	Inclusion	Exclusion
Population	Naturally postmenopausal women	Children and adolescents (<18 years old), premenopausal women, men, animal or in vitro populations
Intervention	HRT with estrogen alone or estrogen + progesterone	Non-hormonal pharmacologic agents used for osteoporosis management (e.g., biphosphonates, denosumab, calcitonin).
Comparator	Exercise therapy, such as weight-bearing exercise	Non-exercise interventions
Outcomes	Changes in BMD, fracture risk reduction, and fall risk	Studies reporting only biochemical markers of bone turnover
Study Design	Peer-reviewed systematic reviews, meta-analyses, randomized controlled trials, and cohort studies with full text available in English published within the last 10 years (2015-2025)	Studies with follow-up <6 months, case reports, editorials, opinion pieces, narrative reviews, conference abstracts without full study data, or published more than 10 years ago (before 2015)

A comprehensive search was conducted from February 24, 2025, to April 5, 2025, across the following databases: PubMed, Embase, Cochrane Library, CINAHL Ultimate, PsycINFO, and MEDLINE (Ovid). The search strategy combined Medical Subject Headings (MeSH) terms and keyword variants for “Hormone Replacement Therapy,” “Exercise,” “Bone Mineral Density,” and “Postmenopausal Women” using Boolean operators. Results were limited to English-language studies published between January 2015 and March 2025. Unpublished studies, dissertations, and grey literature were excluded (Table 2).

**Table 2:** Search Strategy and Results Data derived from search completed on March 6, 2025. Duplicates removal performed using Rayyan (Rayyan Systems Inc., Cambridge, MA, USA). Twenty duplicates were removed (19 deleted, one manually resolved).

Steps	Terms	Databases						
		PubMed	EBSCO: MEDLINE	PsycINFO	Cochrane Reviews (CDSR)	Cochrane Trials (CENTRAL)	EBSCO: CINAHL	Total
		Hits						
1	"Hormone replacement therapy" OR "HRT" OR "estrogen therapy" OR "estrogen replacement therapy" OR "estradiol"	1,72,940	70,897	99	287	18,441	37,895	3,00,559
2	"Exercise" OR "physical activity" OR "weight-bearing exercise" OR "resistance training" OR "strength training" OR "high-impact exercise" OR "aerobic exercise"	4,17,900	10,77,002	1,91,25,926	2,395	1,82,713	2,99,306	2,11,05,242
3	"Bone mineral density" OR "BMD" OR "osteoporosis" OR "fracture risk" OR "bone loss" OR "osteoporotic fractures" OR "bone fragility"	3,55,437	4,51,167	8,92,227	590	22,912	1,22,954	18,45,287
4	"Postmenopausal women" OR "aging women" OR "menopause transition" OR "perimenopause"	52,40,669	1,08,88,638	2,64,908	309	19,416	20,190	1,64,34,130
5	1 AND 2 AND 3 AND 4	447	487	1	27	67	664	1693
6	1 AND 2 AND 3 AND 4 (Limit: English Language Only)	409	441	1	14	17	659	1541
7	1 AND 2 AND 3 AND 4 (Limit: Last 10 Years, 2015-2025)	42	17	-	14	17	243	333
8	1 AND 2 AND 3 AND 4 (Limit: English + Last 10 Years, 2015-2025)	40	16	-	14	17	241	328
9	Duplicates Removed	1	7	-	-	1	10	19

**Figure 1:**
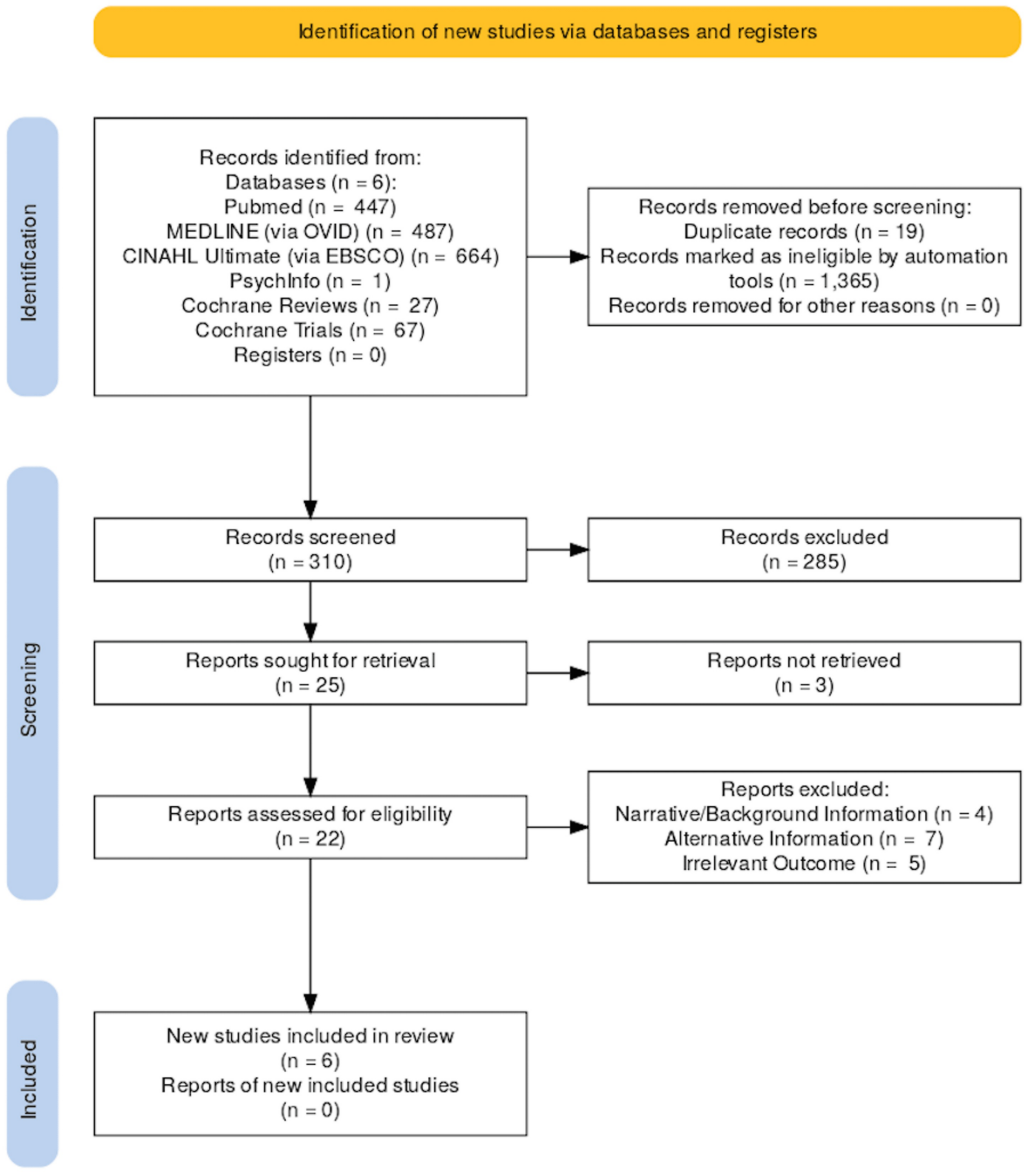
PRISMA Flow Diagram PRISMA: Preferred Reporting Items for Systematic Reviews and Meta-Analyses

A full-text review was conducted independently by two reviewers for all articles that passed the initial screening. Disagreements were resolved through discussion, and studies were grouped into three intervention categories for comparative purposes: HRT-only, Exercise-only, and HRT versus Exercise or Combination. A PRISMA flow diagram summarizes the screening and inclusion process (Figure 1).

Eligible studies included naturally postmenopausal women who were evaluated for changes in bone health following HRT and/or weight-bearing exercise interventions. Studies were required to report at least one primary outcome relevant to bone health, including BMD, fracture risk, or fall risk. Included study designs included randomized controlled trials (RCTs), cohort studies, systematic reviews, and meta-analyses.

Studies were excluded if they enrolled children, men, or premenopausal women or if they focused on nonintervention-based approaches, such as observational assessments of bone turnover markers without a treatment or exercise component. Articles examining pharmacologic therapies other than HRT, such as bisphosphonates or denosumab, were excluded unless HRT was independently analyzed. Additional exclusions included animal or in vitro studies, case reports, editorials, opinion pieces, narrative reviews, and conference abstracts without accessible full data. Studies with follow-up durations of less than six months were also excluded due to insufficient time to evaluate meaningful changes in bone outcomes.

Data was extracted using a standardized Microsoft Excel form (Microsoft Corp., Redmond, WA, USA). The extracted fields included reference and publication year, population characteristics, study design, BMD measurement tools such as dual-energy X-ray absorptiometry (DXA) scans, bone health outcomes, and reported limitations or methodological gaps. Data were organized according to the intervention arm to enable a structured comparison.

Formal risk-of-bias tools were not applied systematically due to the heterogeneity of the study types included. However, quality indicators such as randomization, sample size, and follow-up duration were noted during extraction and integrated into the narrative synthesis. Common sources of bias, such as intervention variability, lack of blinding, or short duration, were highlighted in the descriptive analysis (Table 3).

**Table 3:** Detailed Description of Included Studies BMD: bone mineral density; HT: hormone therapy; E: exercise; PA: physical activity; DXA: dual-energy X-ray absorptiometry; WHI: Women’s Health Initiative; HERS: heart and estrogen/progestin replacement study; CT: controlled trial; RCT: randomized controlled trial; HRT: hormone replacement therapy; FN: femoral neck; LS: lumbar spine

Author, Year	Study Design	Participants and Outcome Measurement	Intervention/Exposure	Comparator	Key Findings	Limitations
Zhao et al. (2015)	Systematic review and meta-analysis of CTs and RCTs. Some studies were RCTs, but not all. No blinding for oestrogen interventions; only one trial had blinding of the measurer	- 764 postmenopausal women, focusing on changes in FN and LS BMD due to combined HRT and exercise interventions versus exercise-only interventions. The primary endpoint is the change in BMD measured by DXA, with data extracted from CTs and RCTs.	- HRT: Transdermal estrogen patches, unopposed estrogen, estrogen plus progesterone taken orally, oral estrogen plus testosterone. Duration: 1 to 5.9 years.	Exercise-only intervention: - Impact Exercise: Jumping, skipping, stepping. Frequency: three times weekly with 50 vertical jumps each time. - Resistance Training: two weekly strength training sessions with two sets of moderate-intensity and three sets of high-intensity resistance exercises. - Mixed Loading Exercise: Combination of impact and resistance training. Activities included jumping, skipping, jogging, walking, stair climbing, and resistance training. Frequency: two to six times per week. Duration: 9 to 18 months.	- The combination of HRT and exercise significantly improved BMD in both the FN and LS compared to exercise alone. - The combination of HRT and exercise was more effective than exercise alone in preventing postmenopausal bone loss. - Mixed-loading exercise protocols were more sensitive to HRT than single-mode exercises in improving BMD.	- Relatively low quality score of included trials - Non-randomized assignment in some trials - Use of per protocol approach instead of ITT analysis - Reliance on DXA for BMD measurements - Limited number of eligible clinical trials - Highly selected samples of postmenopausal women - Need for further studies to explore structural changes and BMD changes
Marjoribanks et al. (2017)	Systematic review and meta-analysis of RCTs that were double-blinded and placebo-controlled with no crossover design	- 43,637 women investigating HT versus placebo for at least one year in perimenopausal and postmenopausal women. - Data sources: HERS 1998 and WHI 1998, Cochrane Gynaecology and Fertility Group Trials Register, CENTRAL, MEDLINE, Embase, PsycINFO. - Outcomes: Mortality, cardiovascular outcomes, cancer, gallbladder disease, fracture, and cognition.	Estrogen, with or without progestin therapy, was administered via oral, transdermal, subcutaneous, or intranasal routes for 12 months or longer (combined continuous HT for 5.6 years and oestrogen-only HT for 7.1 years).	Placebo	- Combined continuous hormone therapy increased the risk of coronary events and venous thromboembolism in relatively healthy postmenopausal women, but was outweighed by the benefit of reduced fracture risk. - Hormone therapy reduced the risk of fractures, which is a significant clinical benefit. - Hormone therapy is not recommended for preventing cardiovascular disease, dementia, or cognitive decline, and should be avoided in women with certain health conditions.	- Limited age representation: Only 30% of women were 50 to 59 years old at baseline. - Insufficient data on long-term HT risks for perimenopausal women and postmenopausal women under 50. - Lack of safety assessment for HT in specific groups (perimenopausal, under 50, temporary/permanent ovarian failure). - Limited understanding of modulating factors (clinical characteristics, biomarkers, different hormones, time frames, doses, routes). - Need for evidence on alternatives to HT for symptom relief.
Akbari et al. (2024)	Systematic review of RCTs	- 506 postmenopausal women (50-75 years old) diagnosed with osteoporosis or osteopenia based on DXA criteria across Europe, Asia, and North America. - Outcomes: BMD measured at the LS, FN, and total hip using DXA in two studies, muscle strength through dynamometer test, 30-s chair stand test, and arm curl in three studies, OP QOL and Qualeffo-41 questionnaires in two studies.	Exercise therapy ranging from 8 weeks to 18 months: - Balance exercises - Strengthening exercises - Stretching exercises - Stability exercises - Motor control exercises - Yoga - Aquatic exercises - Land exercises - Strength exercises - Breathing exercises - Range of motion exercises - Sessions lasting 50 to 60 minutes - Conducted over periods of three weeks to six months - Combined strength and balance training for 40 minutes per session over 20 weeks	Non-exercise control group	- Exercise therapy interventions positively increased bone mass, muscular strength, and quality of life in osteoporotic postmenopausal women. - The effect sizes of these interventions ranged from "small" to "moderate," indicating measurable improvements. - Balance, strengthening, stretching, stability, and motor control exercises were effective in improving bone mass and muscle strength.	- Only studies published in English were considered. - Studies had PEDro scores between 6 and 9, with more than 5 indicating a medium to high quality. This could have introduced bias. - Variability in follow-up periods among studies. - Most studies had brief follow-up periods. - Relatively small sample size.
Sheedy et al. (2023)	Prospective observational study	- 1,342 participants were originally enrolled, 1,026 at the five-year mark appeared for follow-up examination, with 961 postmenopausal women's data analyzed in the end due to missing data on either the PA, BMD, or hormone therapy. Initially, data were determined at baseline from 1993 to 1998, plus the follow-up study conducted five years later from 2002 to 2005. The outcomes assessed were BMD, self-reported daily PA levels, questionnaires on demographics, and risk factors for osteoporosis.	Use of hormone therapy (HT) was the primary intervention. Participants were categorized into three groups based on their HT use: non-users, those who used at baseline but not at year 5, and those who used at baseline and continued use at year 5. No specific PA intervention was implemented. Frequency, duration, and dose of HT are not specified. BMD was assessed with DEXA scanning at baseline and year 5 at the total hip and FN regions.	Continued hormone therapy use	- Bone density decreased in postmenopausal women who discontinued hormone therapy and in those who never used it, while it was maintained in current users. - Usual PA did not mitigate bone loss in women who discontinued hormone therapy. - Targeted PA regimens are suggested for further investigation to potentially mitigate bone loss in older postmenopausal women.	- Observational study design and relatively small sample size - Inability to characterize adherence to prescribed hormone therapy between baseline and year 5. - Age range examined was beyond the immediate postmenopausal period, where the sharpest decline in BMD traditionally occurs. This might have been a missed opportunity to study the early postmenopausal period and the effect PA might have in that window. - Lack of a specific PA regimen for participants.
Satpathy et al. (2024)	RCT with a parallel design	- 150 postmenopausal women aged 50 to 65 years old, at least five years postmenopausal with no history of osteoporosis or bone fractures, divided into three groups: exercise, calcium supplementation, and hormone-replacement therapy. The dataset includes measurements of BMD at the LS and hip regions at baseline and after 12 months.	- Exercise: Structured program with weight-bearing and resistance exercises, three sessions per week, each lasting 60 minutes, for 12 months. - Calcium Supplementation: Daily, 1000 mg of elemental calcium, for 12 months. - HRT: Combination of estrogen and progestin, dosages determined individually based on North American Menopause Society guidelines, for 12 months. Outcome: change in BMD at the LS and FN regions, assessed with DEXA scan.	- Comparator for Group A (Exercise): Group B (Calcium Supplementation) and Group C (Hormone-Replacement Therapy) - Comparator for Group B (Calcium Supplementation): Group A (Exercise) and Group C (Hormone-Replacement Therapy) - Comparator for Group C (Hormone-Replacement Therapy): Group A (Exercise) and Group B (Calcium Supplementation)	- HRT was the most effective intervention at increasing BMD in postmenopausal women, with a mean increase in BMD of 3.2% at the LS and 2.9% at the hip. - Exercise resulted in a mean increase in BMD of 1.5% at the LS and 1.2% at the hip, which was significant, but not as significant as the HRT group. Calcium supplementation showed a mean increase of 1.0% at the LS and 0.8% at the hip, indicating limited efficacy. - HRT significantly reduced bone resorption, as indicated by decreased urinary deoxypyridinoline levels.	- small sample size may limit generalizability - study duration of 12 months may not capture long-term effects - need for future studies with larger sample sizes and longer follow-up periods
Born et al. (2022)	Systematic review and meta-analysis. Included CTs with three study arms: hormone therapy (HT), exercise, and a combination of both (HT + E). Included both randomized and non-RCTs	- 219 in the exercise group, 178 in the HT group, 188 in the HT +E group, and 189 in the control group. These studies were conducted between 1995 and 2007 in Finland, the UK, and the US. Participant characteristics, exercise characteristics, hormone therapy characteristics, and methodological quality assessments were described.	- Hormone therapy (HT): Various compositions and dosages; oral or transdermal application; duration of 11 or 12 months. - Exercise (E): Dynamic resistance training, low-impact weight-bearing exercise, high-impact weight-bearing exercise; frequency of two to six sessions per week; duration of 11 or 12 months; intensity moderate to high. Outcome: BMD via DEXA scan at the LS and FN.	Non-exercise/non-HT control group	- All studies had a positive LS BMD change in the HT+E group, and all HT+E groups and 5/6 exercise groups had positive effects on BMD at the FN. The study found that adding exercise to hormone therapy (HT + E) did not significantly increase BMD at the LS and FN compared to hormone therapy alone. - The effects of HT + E on BMD were slightly higher but not statistically significant compared to isolated hormone therapy. - The study concludes that there is no significant difference in BMD changes between HT + E and HT alone in postmenopausal women.	- A low number of studies were included in the analysis. - Challenges with imputing standard deviations due to missing data. - Heterogeneous hormone therapy supplementation across studies. - Potential dilution of the hormone therapy effect due to participants already on therapy. - Eligibility criteria for control groups might be too short - Variability in exercise programs between trials. - Inclusion of both areal and volumetric BMD measurements. - Limited generalizability due to focus on early-postmenopausal women.

Data Analysis

A narrative synthesis approach was used for this review. No new meta-analysis or meta-regression was conducted because the included studies varied substantially in design, intervention type, duration, and outcome measurements. Study-level statistics such as mean differences, standardized mean differences (SMDs), p-values, and 95% confidence intervals (CIs) were extracted directly from each eligible publication and compared descriptively. Findings were organized into HRT-only, Exercise-only, and Combined-intervention categories to allow comparison of effect direction and magnitude across interventions.

Systematic review

Hormone Replacement Therapy (HRT)

Across the six included studies, both HRT and structured exercise interventions were associated with improvements in BMD among postmenopausal women. In the Cochrane review by Marjoribanks et al., data from the Women’s Health Initiative (WHI) trial showed that estrogen-only HRT significantly reduced the risk of both hip and vertebral fractures over 7.1 years of follow-up (hip: risk ratio (RR) 0.66; 95% CI, 0.46-0.95; P = 0.026; vertebral: RR 0.64, 95% CI, 0.44-0.94; P = 0.021), with absolute risk reductions of five per 1000 women for both hip and vertebral fractures [14]. Combined estrogen-progestin therapy also significantly reduced fracture risk at four to six years (hip: RR 0.67, 95% CI, 0.47-0.96; P = 0.027; vertebral: RR 0.68, 95% CI, 0.49-0.96; P = 0.029), though the effect on vertebral fractures was not sustained at 7.9 years. Importantly, both arms of the WHI trial showed a reduced incidence of any fracture with hormone therapy at both 5.6 and 7.9 years of follow-up. The increase in BMD in the HRT group was greater than the BMD increase in the exercise group and was statistically significant (P < 0.01). All estrogens, with and without progestogens, were administered orally, transdermally, subcutaneously, or intranasally and given for 12 months or longer compared with placebo. The formulation, dose and durations of the uncombined HRT included oral 17-B estradiol 1 mg daily for two or five years, oral estradiol valerate 2 mg daily for two to three years, transdermal estradiol patch with either 0.014 mg/day, 0.025 mg/day, 0.05 mg/day or 0.075 mg/day for two years, intranasal estradiol 0.15 mg or 0.3 mg daily for one to two years, conjugated equine estrogen (CEE) 0.625 mg daily or 1.25 mg daily for a median of 5.8-5.8 years of the intervention and then 7.1-7.9 years follow-up. The formulation, dose and durations of the combined HRT with estrogen and progestogen therapies included continuous combined regimen of CEE 0.625 mg + medroxyprogesterone acetate (MPA) 2.5 mg daily for a mean of 5.6 years and 7.9 year total follow-up, CEE 2.5 mg + MPA 10 mg daily for 10 years, oral estradiol 2 mg + norethisterone 1 mg daily for 2.3 years, estradiol 1 mg daily + MPA 5 mg for 12 days/year for two years, estradiol 1 mg daily for four days per week + 1 mg E2 + 0.35 mg norethindrone three days per week for two years, estradiol 2 mg for days 1-22 + estradiol 1 mg days 23-28 + norethisterone 1 mg days 13-22, estradiol 1 mg + dydrogesterone 5 mg or 10 mg days 14-28 for one year, estradiol 2 mg + dydrogesterone 10-20 mg days 14-28 for one year, estradiol patch 0.05 mg/day + micronized progesterone 200 mg 12 days per month for four years, CEE 0.45 mg daily + micronized progesterone 200 mg 12 days per month for four years, CEE 0.625 mg + MPA 10 mg on days 1-12 for three years, and CEE 0.625 mg + micronized progesterone 200 mg days 1-12 for three years. The standard dose of continuous combined HRT is CEE 0.625 mg + MPA 2.5 mg daily for three to five years [14]. Of note, over 90% of participants in each group completed the study with adherence to the interventions [14]. These findings support the role of HRT use in skeletal protection. Although WHI data are high quality, the broader review was limited by reliance on a small number of large trials, potential attrition bias in smaller studies, and heterogeneity in formulations and populations across trials [14]. The benefits were highlighted especially in women who initiated HRT closer to menopause onset, aligning with the “timing hypothesis” that earlier initiation yields more favorable outcomes [4].

When evaluated independently, HRT and exercise each demonstrated efficacy. Sheedy et al. found that discontinuation of HRT led to a significant decline in BMD in 961 women who were enrolled and returned for a follow-up visit five years later to have their BMD assessed, a medication inventory taken, and completed questionnaires. This study focused on the nonuser and current user status of the participants rather than the specific formulations of the HRT each participant was prescribed. They did, however, note the years since the participant stopped taking hormone therapy if they discontinued it at year 5 (less than one year, one year to under two years, and greater than two years) [15]. At year 5, non-users had a BMD loss at the hip of 0.012 g/cm^2^. Bone loss was lower among participants who were on HRT and discontinued it before year 5 (0.021 g/cm^2^), and there was virtually no change in BMD among those who were HRT users at both time points (P < 0.05 compared with HRT nonusers) [15]. This finding suggested that HRT discontinuation is not neutral and may leave women at an increased skeletal risk.

Additionally, all participants who stopped HRT experienced a BMD loss, but the greatest loss was experienced by those who discontinued HRT earlier - in years 1 to 2 and greater than two years prior to year 5 (0.018 and 0.025 g/cm^2^, respectively) compared with less than one year before year 5 (0.025 g/cm^2^ loss; P < 0.05) This study was limited by the inability to assess adherence to hormone therapy between baseline and follow-up, which may have influenced classification. Additionally, the lack of standardized physical activity intervention and the older average participant age (65.9 years) may have reduced the observed effectiveness of exercise in mitigating BMD loss after HRT discontinuation [15].

Exercise Therapy

When reviewing exercise-only studies for the effects of physical activity in isolation, resistance and weight-bearing training consistently demonstrated improvements in BMD across the hip, spine, and femoral neck (FN) regions. The systematic review by Akbari et al. concluded that exercise therapy was particularly beneficial for women with osteoporosis, contributing not only to improved BMD but also to increased muscular strength and quality of life [16]. They included seven articles, each with sample sizes ranging from 30 to 92 participants (n = 506 participants). The exercise interventions included strength, balance, flexibility, postural stability, movement control, muscle strength, yoga, aquatic, and land exercise with variations in duration and volume [16]. Yoga and aquatic exercises done on land were compared with strength exercises, breathing exercises, range of motion, and balance exercises three times a week, with each session lasting 50 to 60 minutes over 20 weeks [16]. Strength and balance training for 40 minutes each session twice a week for 20 weeks was also investigated. Stretching and strengthening exercises aimed at improving functional level, such as squatting and getting up and down from the ground, were conducted twice a week for 10 weeks [16]. Another group did static balance exercises and upper and lower limb strengthening exercises for 60 minutes three times a week for six months [16]. The systematic review was limited by significant variability in exercise protocols and short study durations in many included trials. Additionally, most studies lacked fracture outcomes and were of lower methodological quality, with limited randomization and blinding [16].

Combined Therapy

In the meta-analysis by Zhao et al., which resulted in six clinical trials with a (n = 764), estrogen therapy enhanced skeletal response to exercise, with combination therapy producing greater increases in BMD than either modality alone, with significance in the FN BMD (SMD = 0.220; 95% CI, 0.01-0.43; P = 0.039) and lumbar spine (LS) BMD (SMD = 0.729; 95% CI, 0.19-1.27, P = 0.009) [17]. They also investigated the combined effect of HRT with different modalities of exercise, such as mixed loading exercise programs and single-mode exercise. They determined that HRT with mixed loading exercise programs prevented postmenopausal bone loss in the spine to a greater extent than HRT with single-mode exercise (SMD = 1.073; 95% CI, 0.14-2.01; P = 0.024). Formulation of HRT was reported as oral conjugated estrogen 0.625 mg/day with MPA 5 mg/day for 13 consecutive days every third month for nine months, and taken for a total of 18 months. Additionally, it also investigated CEE 0.625 mg daily by itself for 12 months. Of note, some of the formulations were not reported [14]. In summary, these findings suggest that while both HRT and exercise are effective, they may offer synergistic benefits when combined. Limitations of the study included a lack of blinding in most studies, the absence of intention-to-treat analysis, and the use of DXA as the only method for assessing BMD [17].

Similarly, Satpathy et al. found that postmenopausal women (n = 150) receiving both HRT and exercise demonstrated greater BMD gains compared to HRT or exercise alone, with a mean increase in BMD of 3.2% at the LS and 2.9% at the hip (P < 0.05) [18]. Satpathy et al. did not provide the formulation, route, or dose of the HRT. Instead, stating that a combination of estrogen and progestin was used, with a specific formulation and dosage determined based on individual needs and risk factors for a duration of 12 months. Their findings were limited by a small sample size, short follow-up of 12 months, and a lack of blinding or control for confounding lifestyle variables [18].

Notably, Born et al. conducted a systematic review with six studies that had sample sizes of eight to 91 participants. They included a subgroup analysis in their meta-analysis of exercise (n = 219), HRT (n = 178), HRT + Exercise (n = 188), and control groups (n = 189) that specifically compared the HRT and exercise arms. The exercise regimens consisted of six sessions per week, with varying intensities and types, including dynamic resistance training (DRT), low- and high-impact weight-based exercises, and mixed protocols. The hormone therapy included combined estradiol 2 mg + norethisterone acetate 1 mg for one year, conjugated estrogen 0.625 mg/d + MPA 5 mg/d for 12 consecutive days every three months, conjugated estrogen 0.624 mg/d daily for 12 months, transdermal estrogen, oral estrogen, and estrogen + testosterone [19]. They determined that both exercise and HRT were effective in preserving BMD, with five out of five HRT + Exercise groups reporting positive changes in LS BMD [19]. However, when comparing HRT + Exercise to HRT alone, there was an insignificant increase in LS-BMD (SMD = 0.19; 95% CI, -0.15 to 0.53; P = 0.27). They also analyzed the effect on LS-BMD when using HRT alone with exercise alone and found it to be mildly in favor of HRT alone (SMD = 0.34; 95% CI, -0.04 to 0.73; P = 0.080). Similarly, in the FN, there was no statistically significant effect when comparing HRT + Exercise with HRT alone (SMD = 0.18; 95% CI, -0.09 to 0.44; P = 0.19). When analyzing HRT alone versus exercise alone in the FN, there was a statistically insignificant effect on BMD in favor of HT (SMD = 0.14; 95% CI -0.38 to 0.65; P = 0.256). This indicates that despite all groups reporting positive changes in BMD, there is not a statistically significant difference in the effect of HRT alone and HRT + Exercise on BMD in the LS or FN. However, this meta-analysis was limited by a small number of included studies (n = 6), missing data requiring imputation of standard deviations, varied hormone therapy regimens, and differences in BMD assessment techniques (DXA vs. quantitative computed tomography (QCT)). Generalizability was also limited to early postmenopausal populations [19].

Broader Limitations and Considerations

Beyond the primary outcomes, these findings align with broader literature suggesting that weight-bearing exercise also supports skeletal muscle mass preservation during postmenopausal weight loss, which is associated with bone loss [6]. Kalogeropoulou highlighted that early postmenopausal weight loss interventions significantly reduce BMD unless accompanied by resistance training, with weight losses of 5-10% associated with approximately 1-2% decreases in bone mass [6]. Studies have also identified key fracture risk factors in this population, including corticosteroid use, lower education level, and high parity [13].

Yang et al., in a cross-sectional study using data from the National Health and Nutrition Examination Survey, found that in 924 postmenopausal women aged 45-65 years (n = 924), greater parity (>6) was associated with lower LS BMD compared to women with lower parity (one to two) (β = - 0.072; 95% CI, -0.13 to -0.02; P = 0.009). In participants with parity greater than or equal to 6, there was a significantly higher prevalence of LS osteoporosis compared to participants with parities of 1 to 2 (OR = 3.88; 95% CI, 1.64-9.18; P = 0.002). This underscores that reproductive history may inform individualized fracture risk assessments (FRAX) [20]. Interestingly, there was no correlation between parity and FN BMD.

In a similar look at individualized approaches to risk assessment, a literature review by Rosso emphasized that there are many alternative treatments, such as phytoestrogen intake, dietary changes, and exercise, that can be used in addition to pharmacologic and lifestyle approaches, particularly among women hesitant to use HRT [12]. While data on skeletal outcomes for these alternatives remains limited, the increasing use of these options highlights the need for ongoing research and personalized patient education [12].

This systematic review is limited by the heterogeneity in study designs, populations, and intervention protocols across the included articles. Some studies included only short-term follow-up, while others did not disaggregate results by fracture site or menopausal age. Additionally, BMD outcomes were not uniformly reported, making direct comparison difficult. There was limited availability of high-quality RCTs directly comparing HRT versus exercise interventions. Finally, we were unable to formally assess the risk of bias across the included studies due to differences in study design and inconsistent reporting of methodological quality.

## Conclusions

HRT and structured exercise interventions, such as weight-bearing or resistance training, are both evidence-based strategies shown to improve BMD in postmenopausal women. When used in combination, these interventions may provide additive benefits, particularly at high-risk fracture sites like the LS and hip. This dual approach may offer the most effective protection against postmenopausal osteoporosis and its related fractures, which are major contributors to morbidity, disability, and mortality among aging populations. The greatest improvement in BMD found by this study was determined to be a 0.8% greater effect at the FN and 2.7% greater effect at the LS with HRT plus exercise in the formulation of oral CEE 0.625 mg/day and MPA 5 mg/day taken for 13 consecutive days every third month for nine to 18 months compared to exercise alone. However, current literature is limited by a small number of direct comparisons between HRT and exercise, inconsistent methods of measuring and reporting BMD outcomes, and limited long-term follow-up. To address these gaps, future research should include RCTs or long-term cohort studies that explore the duration, intensity, and timing of these therapies and how they impact BMD over time. Clinical decision-making should emphasize individualized treatment plans that consider patient-specific factors such as fracture risk, hormonal status, parity, comorbidities, and personal preferences. Tools like the FRAX and patient-shared decision-making may support clinicians in tailoring therapy. For women who cannot or choose not to use HRT, non-hormonal and lifestyle-based options, such as acupuncture, resistance training, or dietary interventions, remain important considerations that need further research into their effects on bone health.

Finally, improving patient and clinician education about the risks and benefits of HRT, particularly in the context of fracture prevention when initiated within the appropriate therapeutic window, remains critical. This review provides a comparative framework to guide personalized treatment decisions and enhance bone health in postmenopausal women.
